# Untargeted Metabolomics of *Alternaria solani*-Challenged Wild Tomato Species *Solanum cheesmaniae* Revealed Key Metabolite Biomarkers and Insight into Altered Metabolic Pathways

**DOI:** 10.3390/metabo13050585

**Published:** 2023-04-24

**Authors:** Dhananjaya Pratap Singh, Mansi Singh Bisen, Ratna Prabha, Sudarshan Maurya, Suresh Reddy Yerasu, Renu Shukla, Jagesh Kumar Tiwari, Krishna Kumar Chaturvedi, Md. Samir Farooqi, Sudhir Srivastava, Anil Rai, Birinchi Kumar Sarma, Nagendra Rai, Prabhakar Mohan Singh, Tusar Kanti Behera, Mohamed A. Farag

**Affiliations:** 1 ICAR-Indian Institute of Vegetable Research, Varanasi 221305, India; 2 ICAR-Indian Agricultural Statistics Research Institute, Library Avenue, New Delhi 110012, India; 3 Indian Council of Agricultural Research, New Delhi 110012, India; 4 Department of Mycology and Plant Pathology, Institute of Agricultural Sciences, Banaras Hindu University, Varanasi 221005, India; 5 Pharmacognosy Department, College of Pharmacy, Cairo University, Cairo 11562, Egypt

**Keywords:** metabolite profiling, secondary metabolites, metabolomics, biotic stress, *Solanum cheesmaniae*, *Alternaria solani*, early blight

## Abstract

Untargeted metabolomics of moderately resistant wild tomato species *Solanum cheesmaniae* revealed an altered metabolite profile in plant leaves in response to *Alternaria solani* pathogen. Leaf metabolites were significantly differentiated in non-stressed versus stressed plants. The samples were discriminated not only by the presence/absence of specific metabolites as distinguished markers of infection, but also on the basis of their relative abundance as important concluding factors. Annotation of metabolite features using the *Arabidopsis thaliana* (KEGG) database revealed 3371 compounds with KEGG identifiers belonging to biosynthetic pathways including secondary metabolites, cofactors, steroids, brassinosteroids, terpernoids, and fatty acids. Annotation using the *Solanum lycopersicum* database in PLANTCYC PMN revealed significantly upregulated (541) and downregulated (485) features distributed in metabolite classes that appeared to play a crucial role in defense, infection prevention, signaling, plant growth, and plant homeostasis to survive under stress conditions. The orthogonal partial least squares discriminant analysis (OPLS-DA), comprising a significant fold change (≥2.0) with VIP score (≥1.0), showed 34 upregulated biomarker metabolites including 5-phosphoribosylamine, kaur-16-en-18-oic acid, pantothenate, and *O*-acetyl-L-homoserine, along with 41 downregulated biomarkers. Downregulated metabolite biomarkers were mapped with pathways specifically known for plant defense, suggesting their prominent role in pathogen resistance. These results hold promise for identifying key biomarker metabolites that contribute to disease resistive metabolic traits/biosynthetic routes. This approach can assist in mQTL development for the stress breeding program in tomato against pathogen interactions.

## 1. Introduction

Chemical diversity and metabolite composition of plants exposed to biotic challenges in the environment critically define their standing status under the conditions of the progression of pathogen infection [1]. Following pathogen invasion, pathophysiological and molecular cascades are triggered to sense the stress [2], which simultaneously activate an integrated cellular network of physiological, biochemical, and molecular mechanisms [3,4]. Further development of pathogen invasion and disease progression starts with the exchange of signaling molecules [5] and chemical crosstalk [6] as a ping-pong mechanism in the host plant and pathogen interaction [7]. Accordingly, a complex cellular and metabolic response is activated inside the host plant against pathogen invasion to circumvent disease progression. As the infection progresses, metabolic networks of the pathogens and host become intertwined, thereby leading to mutually influenced metabolism in both organisms [8]. In plants, an array of metabolites and their intermediates are biosynthesized, typically to either accumulate or become degraded to categorically avoid or restrict pathogen invasion [9]. In order to protect themselves from pathogens, plants need to accumulate a diverse array of primary and secondary metabolites that warrant distinct developmental and functional protective roles [10]. These metabolites, if mapped in pathogen-challenged and unchallenged plants in an integrated way, can establish their identity and functional role and provide exclusive clues regarding plant responses toward pathogens.

*Solanum cheesmaniae* is one of the two main wild tomato species from the Galápagos Islands with enhanced plant performance against stresses [11]. Because of its potential stress-tolerance and disease-resistance traits [12], this species has successfully been used in stress-resistance breeding programs for the improvement of commercial tomato varieties [13]. Early blight-resistant sources have been identified in different wild tomato species, with the accession WIR3928 belonging to *S. cheesmaniae* showing resistance to early blight throughout the vegetative phase and moderate resistance at the flowering stage [14]. Being a wildtype, the plant is supposed to possess stringent metabolic and molecular buildup of metabolic events that strongly activate defense mechanisms. Such metabolic buildup possibly makes plants resistant against invasion by pathogens such as *Alternaria solani* (causing early blight), a devastating pathogenic disease causing >50% damage to cultivated tomato crop under field conditions [15]. For comparatively mapping such chemical buildup inside plant cells and tissues, metabolomics has recently emerged as a practical analytical and informatics tool to offer unbiased qualitative and quantitative screening of metabolites at different status [16]. It was, therefore, hypothesized that the robust intrinsic metabolic fitness of *S. cheesmaniae* can offer the plant overprotection against *A. solani* pathogen. In this study, untargeted metabolomics analysis of *S. cheesmaniae* plants grown under normal and pathogen-challenged conditions was attempted to aid in identifying significantly distinct metabolic biomarkers and biosynthetic clues linked to plant tolerance responses against *A. solani*. The study holds promise for developing disease-diagnostic tools based on key biomarker metabolites, which could lead to the development of mQTLs to target disease-resistance metabolic traits/biosynthetic routes for supporting speed breeding programs in tomato against biotic stresses.

## 2. Materials and Methods

### 2.1. Plant Material and Growth Conditions

*S. cheesmaniae* accession WIR3928 (wild species moderately resistant to early blight) was used in this study to assess the post-inoculation metabolomic response toward *A. solani* (Indian Type Culture Collection (ITCC) 4632, ICAR-IARI, New Delhi, India). Seeds were surface sterilized using 2% sodium hypochlorite solution for 5 min using agitation followed by washing with sterilized water and drying on sterile filter paper. Dried seeds were seeded inside plastic pots with dimensions 20 × 20 × 14 cm containing a sterilized mixture of field soil, cocopeat, perlite, and vermiculite in a ratio of 3:1:1:1 (*w*/*w*), and then left in greenhouse to grow for 3 weeks. Afterward, seedlings were transplanted in 10 pots containing similar soil substrates. At the early flowering stage (60 days), lower leaves of 10 potted plants were spray-inoculated with the pathogen (2.1 × 10^4^ CFU/mL inoculums). Pots were kept under high relative humidity (RH 86–90%) for 24 h, which was further reduced to 70% for the next 5 days to allow for disease development. The disease incidence (%) was calculated from days 1 to 5 as reported by Yerasu et al. [14]. On the fifth day post inoculation, sampling of upper leaves was performed separately from the pathogen-challenged and non-inoculated plants.

### 2.2. Leaf Sample Preparation and Metabolite Extraction

A comprehensive method was employed to capture both polar and nonpolar metabolites of tomato leaves [17]. Leaf tissues frozen with liquid nitrogen were pulverized with an ice-cold mortar and pestle. Five grams of leaves from pathogen-challenged and unchallenged plants were extracted using ethyl acetate (100%, 20 mL) overnight to capture nonpolar to moderately polar metabolites [18]. The supernatant was separated using centrifugation (6000× *g*, 4 °C, 15 min) and dried on a water bath set at 55 °C to yield 1 mL of ethyl acetate extract. Tissues were further extracted with methanol and water/methanol (10 mL each, 1:1, *v*/*v*) separately using sonication for 15 min and then vortexed for 2 h. The mixture was kept overnight, and tissue debris was then pelleted using a benchtop centrifuge (6000× *g*, 4 °C, 15 min) to yield 1 mL of each extract using a rotary evaporator (55 °C). All three extracts were pooled together and transferred to a 5 mL glass tube to obtain dried extract using rotavapor at 55 °C for 1 h. The samples were finally reconstituted in 1 mL of HPLC-grade 100% methanol as solvent, filtered through a 0.22 µm syringe filter, and transferred into chromatographic vials, which were capped and stored at 4 °C for further analysis. Three biological replications for each sample were prepared and analyzed in triplicate. The overall results represented two independent experiments. A quality check of the samples in aliquots was also performed to monitor sample stability, as well as instrument and analysis deviation. The extraction protocol standards were followed as per the minimum reporting standards explained for chemical analysis [19,20].

### 2.3. Untargeted LC–MS Analysis

A reconstituted extract of tomato leaves (200 µL) was filtered using 0.22 µm syringe filters and analyzed on a Dionex Ultimate 3000 (Thermo Fisher Scientific, Waltham, MA, USA) HPLC system coupled to a Q Exactive mass spectrometer (Thermo Fisher Scientific, USA). The analysis was performed using a Hypersil Gold C18 (2.1 mm × 100 mm, 1.9 µm) column set at 35 °C throughout the sample run of 31 min. The injection volume was 15 µL per sample. The mobile phase was A (0.05% formic acid in water) and B (0.05% formic acid in acetonitrile), with a flow rate of 350 µL/min. The elution conditions were 5% solvent B for 0 to 2 min, followed by 5–95% solvent B for 2.01–22 min, 95% solvent B for 22.01–27 min, and 5% solvent B for 27.01–31 min.

The Q Exactive MS system was used for data acquisition. The full scan MS range was set at 100–1500 *m*/*z*, and the first-order resolution was set at 140,000. Data acquisition was obtained in positive and negative ionization mode with an AGC target of 1 × 10^6^. The parameters for the ion source were set as follows: sheath gas flow rate at 60 (arbitrary unit, au), aux gas flow rate at 20 (au), and sweep gas flow rate at 10 (au). A capillary voltage of (+) 3.2 kV was applied at a capillary temperature of 275 °C. The S-Lens level was at 55 rf, and the probe and aux gas heat temperatures were at 250 and 350 °C, respectively. Each analysis was performed in three technical replicates. A quality check (QC) sample was run after every six samples to ensure accurate retention time and elution order of the HPLC system. The product ion scan was obtained using first- and second-level MS data acquisition mode. The data obtained from LC–MS were analyzed using Compound Discoverer 3.3 (Thermo Fisher Scientific, USA).

### 2.4. Tandem MS Data Processing and Statistical Analysis

Tandem mass spectrometry (MS/MS) parameters were set to improve the mass fragmentation pattern, with an *m*/*z* scan range of 200–2000, isolation offset of *m*/*z* 0.5, collision energy (CE) of 25–45 eV, maximum IT of 50 ms, loop count of 5, MSX count of 1, resolution of 35,000, one microscan, and isolation window of *m*/*z* 2.0. The maximum AGC target for data-dependent acquisition (DDA) was set at 8.00 e^3^ with unassigned charge exclusion having a dynamic exclusion rate of 10 s. An automated selection of precursor ions from an accurate mass inclusion list was prioritized for obtaining MS2 spectra as pre-annotated features after injection of two blanks and three quality checks (QCs) during system conditioning [21]. 

The raw data obtained from LC–MS/MS was preprocessed and analyzed using Compound Discoverer 3.3 (Thermo Fisher Scientific, USA). The analysis primarily included peak extraction, intensity detection, retention time (r.t.) correlation, filling of missing values, adjoint ion combinations, peak alignment, and normalization. The processing further facilitated metabolite identification and detection of molecular weight, retention time, and peak area. Metabolite features (*m*/*z*) were annotated using four chemical databases, namely, mzCloud (www.mzcloud.org), predicted compositions (www.thermofisher.com), metabolicatlas (metabolicatlas.org), and chemspider (www.chemspider.com) (accessed on 14 February 2023). In the absence of authenticated chemical compounds, *m*/*z* features were further reconfirmed and assigned KEEG identifier IDs after their annotation with the KEGG compound database (www.genome.jp/kegg/compound) and PLANTCYC (http://plantcyc.org) following minimum reporting standards (accessed on 14 February, 2023) [19,20].

The data were preprocessed before performing multidimensional statistical analysis, and the missing values were excluded from the original dataset. The processed data were then tabulated in MS Excel and then uploaded to the “Statistical Analysis” module of MetaboAnalyst 5.0 (www.metaboanalyst.ca, accessed on 14 February 2023). Univariate analysis included feature analysis by two-sample *t*-test and Wilcoxon rank-sum tests, fold change (FC) analysis to identify up- and downregulated metabolite features, volcano plot, and a correlation heatmap that aided in the verification of significantly different features. Before performing PCA model, log_2_ transformation was followed by Pareto scaling for scaling the obtained data. Chemometric analysis, including principal component analysis (PCA), partial least squares discriminant analysis (PLS-DA), and orthogonal PLS-DA (OPLS-DA), was performed. The VIP score obtained in the OPLS-DA model and fold-change maxima facilitated the assessment of the influence of intensity and explanatory ability of each of the metabolites in classifying and discriminating groups of samples on the basis of biologically significant metabolite features. Enrichment analysis was performed using MetaboAnalyst 5.0 by annotating KEGG IDs with “main-class” and “sub-class” metabolite chemical sets. Pathway and network analyses were performed.

A larger VIP score (≥1) with fold change (FC) ≥2 showed a high contribution of metabolite features in the sample differentiation in OPLS-DA analysis. The features with VIP ≥ 1 and FC ≥ 2 at *p* ≤ 0.05 were considered “significantly different metabolites” [21] characterized as “biomarkers”. KEGG MAPPER was used to perform functional pathway annotation using “Compound Search” with major metabolic pathways to which the “annotated metabolites” or “differential metabolite biomarkers” belonged to.

### 2.5. Statistical Analysis

The “statistical module” of MetaboAnalyst 5.0 was used for univariate and multivariate data analysis. For other purposes, data were analyzed using SPSS 16.0 for one-way analysis of variance (ANOVA) using Student’s *t*-test.

## 3. Results

**Figure 1:**
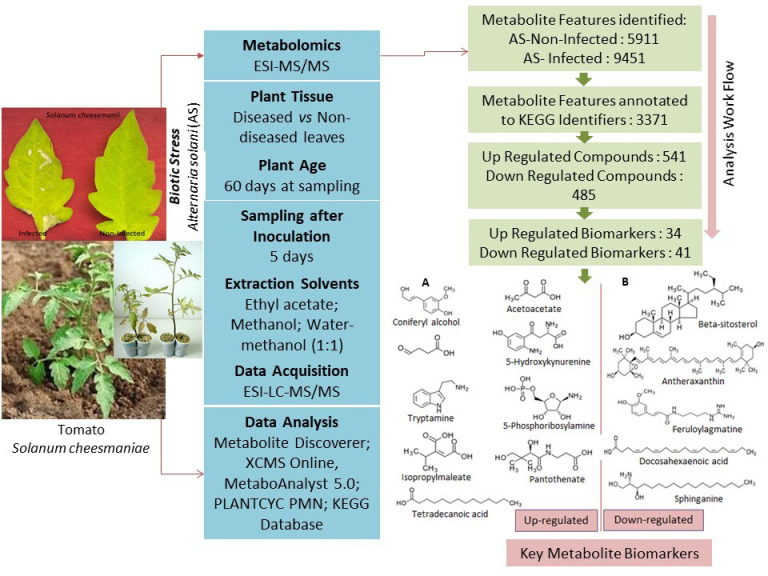
Representative scheme of the experimental setup.

A major goal of this study was to employ untargeted metabolomics to decipher metabolic responses of *S. cheesmaniae* plants against *A. solani* pathogen. The experimental setup, disease incidence, analysis procedure, and the outcomes in brief are summarized in Figure 1. *A. solani* led to a disease incidence of 17.6% based on leaf spot count after 5 days of pathogen inoculation, after which the disease did not spread intensively. LC–MS/MS-based untargeted metabolite profiling of pathogen (*A. solanii*)-challenged (MCTR) and unchallenged (MCNTR) *S. cheesmaniae* plant leaves led to the detection of 10,943 metabolite features. The analysis by “pathway hits” resulted in 92 metabolic pathways (20 at *p* ≤ 0.05) mostly involved in the biosynthesis of ubiquinones, terpenoid-quinones, glycosphingolipids, flavonoids, sesqui-di- and tri-terpenoids, carotenoids, steroids, phenylpropanoids, zeatin, folate, anthocyanins, cutin and suberin, sphingolipid, porphyrins, linolenic acid, glutathione, vitamin B6, thiamine, inositol phosphate, cysteine, methionine, and ascorbate, as well as carbon fixation. Furthermore, functional analysis using “compound hits” matched 3371 compounds with the “metabolite features” having “KEGG identifiers”, along with their matched forms and mass differences (Supplementary Table S1).

### 3.1. Enrichment Analysis

**Figure 2:**
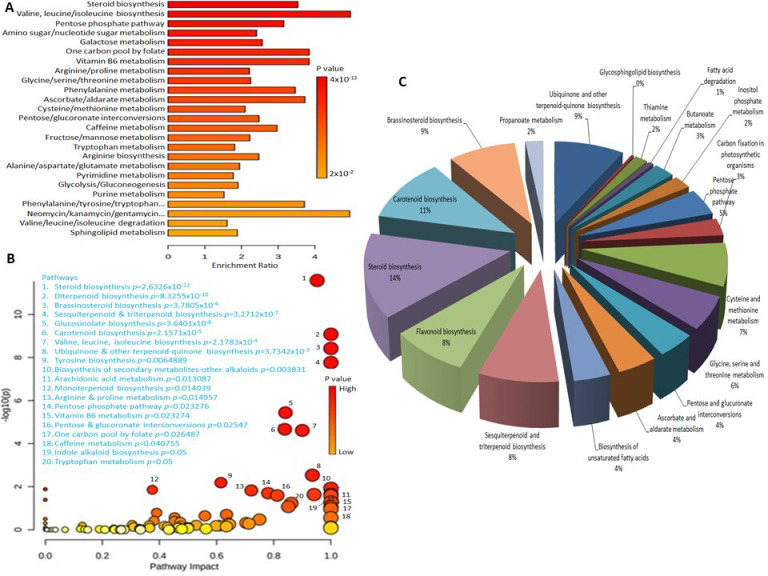
(**A**) Enrichment analysis of metabolite features with KEGG IDs. (**B**) Integrated pathway activity profile of significant (*p* ≤ 0.05) metabolite features linked to biosynthetic pathways as analyzed by MetaboAnalyst 5.0 (GSEA algorithm) using default parameters and the *Arabidopsis thaliana* pathway as the library. (**C**) Significantly enriched major pathways. Enrichment ratio = observed hits/expected hits.

All 3371 metabolite features having KEGG identifiers were annotated with the *Arabidopsis thaliana* pathway library using the “Functional Analysis” module of MetaboAnalyst 5.0. This resulted in the identification of the top 25 enriched metabolic pathways in which metabolite features were significantly linked (*p*-values: 4 × 10^−2^ to 4 × 10^−13^) (Figure 2A). The pathway analysis module further identified 20 significantly enriched metabolic pathways (Figure 2B) belonging to steroid/terpenoid biosynthesis, amino-acid biosynthesis and degradation, and metabolism of arachidonic acid, tryptophan, and sphingolipids. The integrated pathway activity profile of the metabolite hits linked to different pathways after annotation against the *Arabidopsis thaliana* library in KEGG also verified the appropriateness of the functional analysis approach of metabolic features and their pathways (Figure 2C).

### 3.2. Univariate and Multivariate Data Analyses

**Figure 3:**
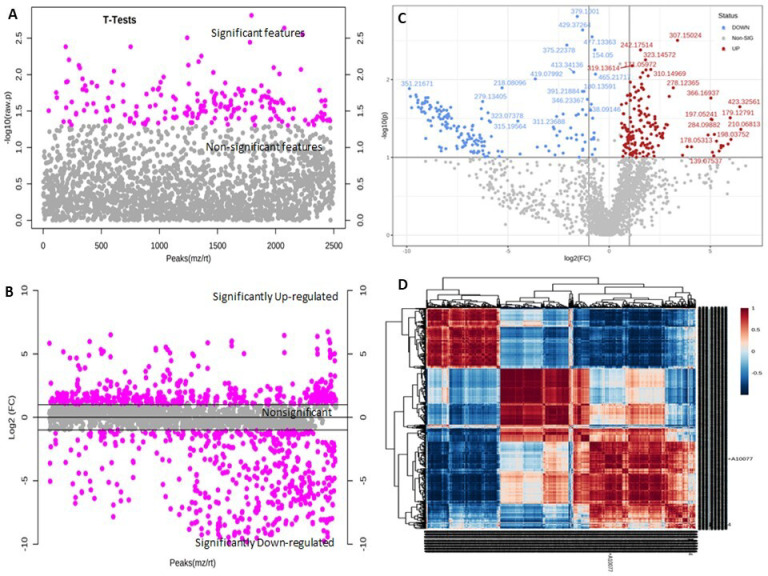
(**A**) Feature analysis by two-sample *t*-test and Wilcoxon rank-sum tests at *p* ≤ 0.05 revealed 201 significant versus 2298 nonsignificant features. (**B**) Fold change (FC) analysis of metabolite features after the plants were exposed to pathogen. At the FC threshold of ≥2.0, 541 significantly upregulated, 485 downregulated, and 1472 nonsignificant features were identified. (**C**) Volcano plot indicating clear differentiation of metabolite features in the two samples. (**D**) Correlation heatmap showing correlation of the top 1000 features based on their interquartile range (IQR) in a large dataset. The distance measured is based on Pearson r values with default MetaboAnalyst parameters.

**Figure 4:**
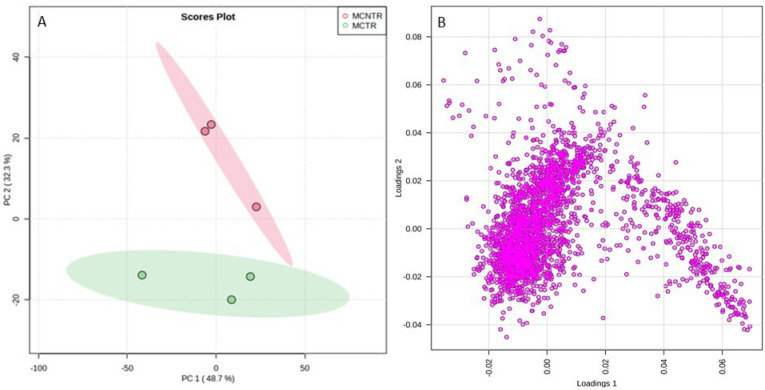
(**A**) Principal component analysis score plot reflecting visualization of the relationship among the samples in terms of groupings, trends, or outliers and showing differences between MCTR and MCNTR along *x*-axis (PC1) and *y*-axis (PC2). Principal components 1 and 2 explained 48.7% and 32.3% of the variance, respectively. (**B**) Loading plot describing the influence of variables in sample segregation.

**Figure 5:**
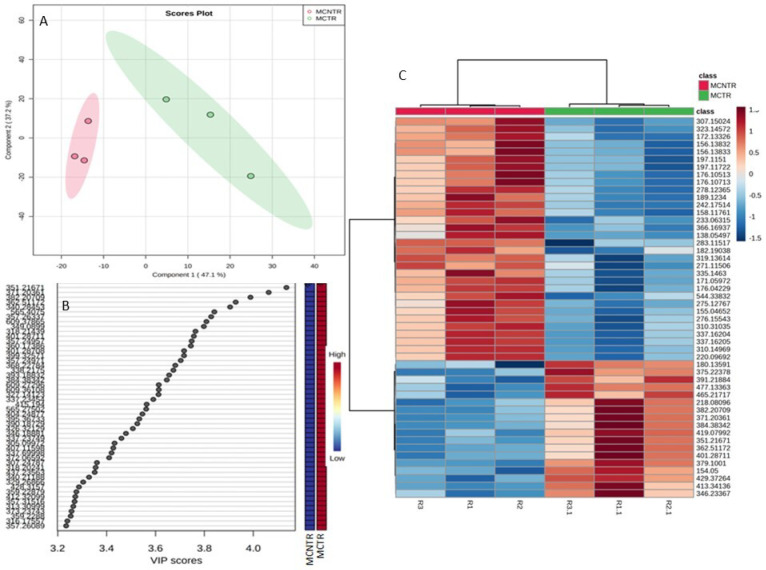
(**A**) PLS-DA score plot with scores (component 1 (34%) versus component 2 (42.3%) plotted from the metabolite profiles of MCTR/MCNTR samples. Both the disease-challenged (MCTR) and the unchallenged (control) (MCNTR) plants were grown under control conditions (*n* = 6) before metabolite extraction and analysis using ESI-MS/MS. The separation of both samples indicated consistent patterns in the metabolites which could explain differences between the diseased and non-diseased groups. (**B**) Top important features with variable importance plot (VIP). A higher VIP score for metabolites represents their importance in influencing the score most. On the Y-axis, variables are ranked in terms of their importance (variables of highest importance at the top). (**C**) Hierarchical clustering of feature abundance normalized by Pareto scaling and *t*-test from the normalized dataset, with Pearson as the distance measure and Ward as the clustering method. The top 50 features are shown as representative clusters. The relative abundance of the features in samples is represented by color (lower (blue) to higher (red) abundance) presented in the key. The brightness in color reflects the differential magnitude when compared with the average value. Dendrograms to the top and left show hierarchical relationships between diseased (MCTR) and non-diseased (MCNTR) samples (**top**) and identified metabolite features (**left**).

Metabolite feature analysis by *t*-test at *p* ≤ 0.05 showed 201 significant features (Figure 3A). Fold change (FC) analysis (threshold FC ≥ 2.0) identified 541 significantly up- and 485 significantly downregulated metabolite features (Figure 3B). Clear differentiation of up- and downregulated significant metabolite features on both axes was visualized using a volcano plot (Figure 3C). A correlation heatmap correlated the top 1000 metabolite features based on the interquartile range (IQR) (Figure 3D). Multivariate data analysis of samples using an unsupervised PCA score plot revealed 48.7% variance along PC1 versus 32.3% variance along PC2, indicating a clear differentiation of metabolite features in MCTR and MCNTR (Figure 4A). The PCA loading plot (Figure 4B) identified the 10 most prominent metabolite features, with *m*/*z* 549.3029, 213.1599, 371.7534, 570.9134, 393.2858, 437.2661, 481.9997, 225.1099, 279.1377, and 205.0708 negatively contributing to PC1. In contrast, the top 10 features with *m*/*z* 374.1479, 281.1897, 391.263, 359.11, 283.1875, 377.0567, 314.1896, 284.1707, 492.3683, and 390.0878 positively contributed to PC1 at the 95% confidence level. Likewise, for PC2, the 10 prominent positively contributing features were *m*/*z* 423.32561, 532.43925, 193.14975, 610.54041, 354.17554, 198.03752, 178.05313, 625.26542, 532.4209, and 533.10217. Negatively contributing metabolite features in PC2 included *m*/*z* 360.17386, 340.28453, 415.194, 349.0899, 565.4075, 384.33342, 362.51172, 371.20361, 351.21671, and 382.20709. The metabolite features screened for supervised PLS-DA also showed clear differentiation of the two samples with component 1 (*x*-axis) covariance at 47.1% and component 2 covariance (*y*-axis) at 37.2% (Figure 5A). The variable importance in projection (VIP) scores resulted in the top 50 features having VIP scores ≥1 in PLS-DA model, which indicated high contribution of the feature metabolites in sample segregation (Figure 5B). Hierarchical clustering showed distinct patterns associated with metabolic changes in MCTR and MCNTR (Figure 5C). Dendrograms showed hierarchical relationships between pathogenic and nonpathogenic conditions and identified key metabolite features. One group of clusters was associated with the plant responses in control, while the other cluster showed high distinction from the nonpathogenic plant groups, demonstrating differential metabolic reprogramming in the pathogen-challenged *S. cheesmaniae* leaf samples.

**Figure 6:**
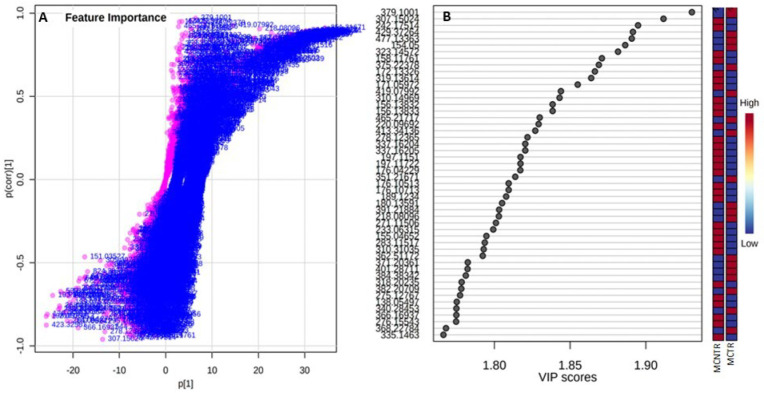
(**A**) OPLS-DA S-Plot (BoxPlot) facilitating visualization of the variable influence in an orthogonal PLS-DA model combining the covariance and correlation loading profile of the metabolite features in the MCTR/MCNTR samples. The plot identifies putative biomarkers (**bottom left** and **top right**) responsible for the separation of groups. (**B**) Variable importance in projection (VIP) scores identifying mass-to-charge ratio (*m*/*z*) with discrimination between sample groups. A higher VIP score denotes a better influence of the metabolite feature in discriminating between the two groups. The mini heatmap on the right indicates the concentration variations within the two groups.

The discrimination of metabolite level between control and challenged plants was further revealed using an OPLS-DA loading S-plot, which highlighted relevant feature ions with high covariance to act as discriminatory biomarkers (Figure 6A). The “outlier” feature ions in the top right quadrant were positively correlated, while those in the lower left quadrant were negatively correlated with the response of the plants under pathogenic conditions. The features were further validated using a VIP plot with VIP scores (Figure 6B) at values ≥1.

### 3.3. Pathway Classification of Annotated Features by KEGG Mapper

**Figure 7:**
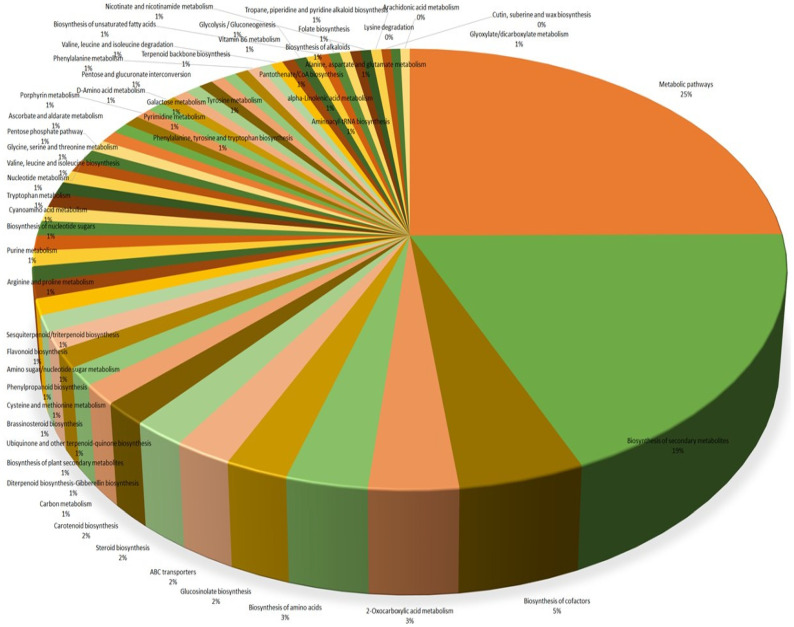
Enrichment pathway of the up- and down-regulated compounds in pathogen-challenged and unchallenged (control) tomato samples.

All chemical query identifiers (3371) obtained from the functional annotation of the metabolite peak list when mapped against the *S. lycopersicum* database in KEGG Mapper revealed 107 pathways and 173 modules. As many as 530 compounds were mapped with metabolic pathways (sly01100): 449 related to biosynthesis of secondary metabolites, along with 25 to phenylpropanoids, 100 to cofactors, 71 to 2-oxocarboxylic acid metabolism, 31 to carbon metabolism, 65 to amino acids, 46 to ABC transporters, 42 to steroids, 36 to carotenoids, 28 to diterpenoids, 27 to ubiquinone and terpenoids/quinones, and 24 to sesquiterpenoid/triterpenoid and flavonoid biosynthesis (Figure 7). In addition to major metabolic pathways routinely involved in plant growth, a large set of metabolite features were mapped with those compounds which were directly linked to secondary metabolite biosynthesis, which have been shown to exert a direct role in the defense of plants challenged with the pathogen.

### 3.4. Enrichment Analysis of Up- and Downregulated Compounds

**Figure 8:**
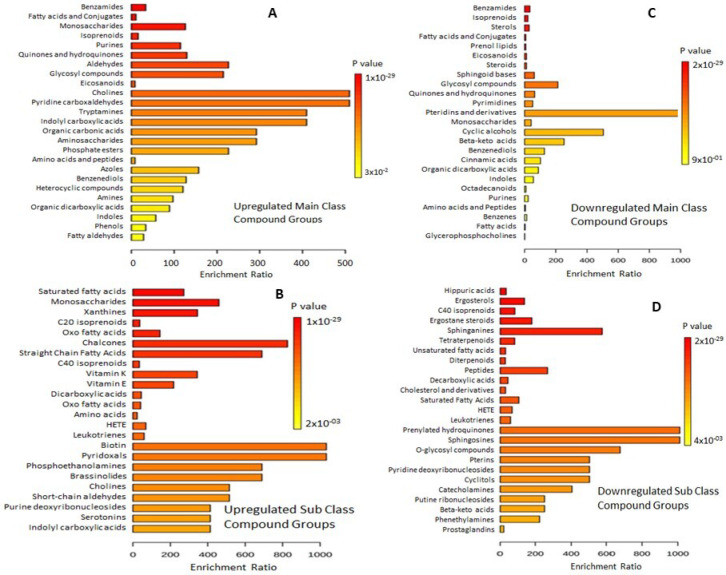
Metabolite sets: overview of up- and down-regulated compounds in the metabolic pathways of tomato. (**A**) up-regulated main class compound groups; (**B**) up-regulated sub-class compound groups; (**C**) down-regulated main class compound groups and (**D**) down-regulated sub-class compound groups. Annotation was performed with 464 main chemical class metabolite sets and 1072 sub chemical class metabolite sets.

FC analysis resulted in 541 significantly up- and 485 significantly downregulated features at FC ≥ 2.0. These features, when matched with the *S. lycopersicum* (and *S. pinnelli*) compound database in PLANT CYC PMN, revealed 179 features having KEGG IDs for upregulated and 175 features with KEGG IDs for downregulated compounds. In KEGG Mapper, the upregulated compounds were mapped with 77 pathways and 61 modules, while downregulated compounds were mapped with 88 pathways and 80 modules. The main classes of upregulated classified compound groups to which the metabolites belonged were fatty-acid conjugates, aldehydes, cholines, tryptamines, indolyl carboxylic acid, indoles, and phenols in the range of *p*-values 3×10⁻^2^ to 1×10⁻^29^ (Figure 8A). The subclass compound groups included chalcones, straight-chain fatty acids, vitamins K and E, biotic, pyridoxals, phosphoethanolamines, and serotonins (*p*-value range: 2×10⁻^3^ to 1×10⁻^29^) (Figure 8B). Similarly, the most prominent downregulated main class compound groups were derivatives of cyclic alcohols and beta-keto acids (*p* = 9×10⁻^1^ to 2×10⁻^29^) (Figure 8C). The subclass of downregulated compound groups belonged to sphinganines, ergostane steroids, peptides, prenylated hydoquinones, sphingosines, catecholamines, and phenethylamines (Figure 8D).

**Figure 9:**
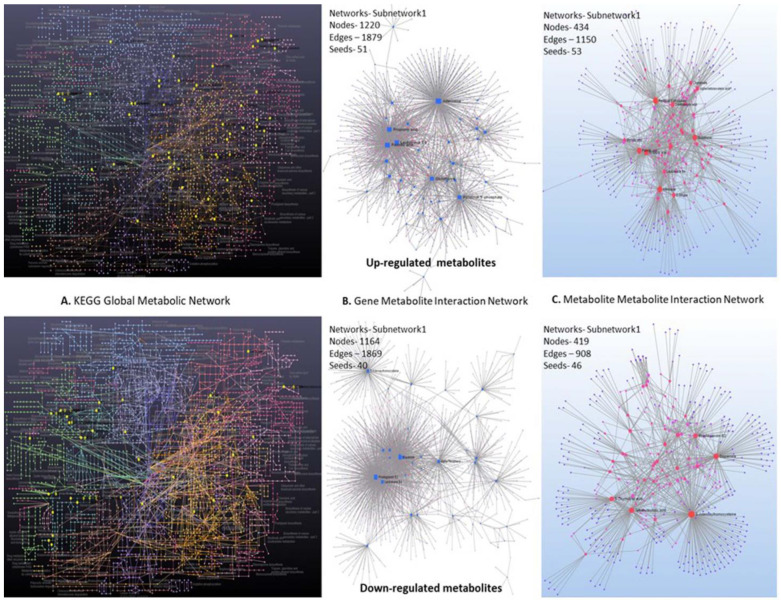
Network analysis of up- and down-regulated compounds in diseased and non-diseased tomato samples based on KEGG Global metabolic network: gene–metabolite interaction and metabolite–metabolite interaction.

The results clearly reflected differentiation among the up- and down-regulated compound groups. It was confirmed that different metabolites differentially contributed to the metabolic pathways to influence biosynthesis of compounds in favor of plant responses toward pathogen challenges. Up- and down-regulated metabolites having KEGG identifiers were mapped and visualized in KEGG global metabolic network (Figure 9A) that showed compounds in the pathways. The gene–metabolite interaction network explored interactions between the functionally related metabolites and genes (Figure 9B). The input metabolites and genes (seeds) were mapped to the selected interaction network that created subnetworks 1 and 2 with 1220 and five nodes, 1879 and four edges, and 51 and two seeds (genes) for upregulated compounds. The metabolite–metabolite interaction network highlighted potential functional relationships between annotated metabolites in up- and downregulated compounds (Figure 9C). Upregulated compounds were networked with 434 nodes, 1150 edges, and 53 seeds (genes), while downregulated compounds showed 419 nodes, 908 edges, and 46 seeds (genes).

### 3.5. Potential Upregulated Metabolites

Bioactive compounds including trans-cinnamate (C00423; *m*/*z* 146.03781), a tetrahydrofuran lignin, pinoresinol (C05366, *m*/*z* 359.13143), and 5-hydroxyconiferyl alcohol (C12205, *m*/*z* 241.07068) were identified as the metabolites involved in phenylpropanoid biosynthesis. Increased abundance of flavonoids, the prominent defense-indicator antioxidant compounds to impart improved plant resistance, was found in pathogen-challenged plant leaves, A range of upregulated metabolites involved in flavonoid biosynthesis including phloretin (C00774, *m*/*z* 275.07353), pinocembrin (5,7-dihydroxyflavanone) (C09827, *m*/*z* 257.06297), pinocembrin chalcone (C16404, *m*/*z* 257.06299), apiforol (C12124, *m*/*z* 275.07355), isoliquiritigenin (C08650, *m*/*z* 257.06298), and deoxyleucopelargonidin (C16415, *m*/*z* 275.07357) were identified. Carotenoids such as canthaxanthin (C08583, *m*/*z* 579.38672), zeaxanthin (C06098, *m*/*z* 283.20569), phoenicoxanthin (C15967, m/z 579.38674), 9’-cis-neoxanthin (C13431, *m*/*z* 615.40351), violaxanthin (C08614, *m*/*z* 615.40353), and xanthoxin (C13453, *m*/*z* 290.17496) were significantly upregulated in *S. cheesmaniae* following pathogen challenge.

Coniferyl alcohol (C00590, *m*/*z* 220.09692) involved in lignin biosynthesis was up-regulated along with oxlipids, i.e., leukotriene A4 (C00909, *m*/*z* 317.21106) and icosapentaenoic acid (C06428, *m*/*z* 283.20569). Furthermore, short-chain alcohols and aldehydes, e.g., 3-hexenol (C08492, *m*/*z* 145.08606) and 3,6-nonadienal (C16323, *m*/*z* 197.11722), a post-infection hypersensitive response inducer in plants, were upregulated. Phytohormone derivative abscisic aldehyde (C13455, *m*/*z* 247.13288) and variants and intermediates of GA including GA A9 (C11863; *m*/*z* 353.13428), A20 (C02035, *m*/*z* 353.13582), A4 (C11864), and A51 (C11865, *m*/*z* 353.13583) were identified in pathogen-challenged plant leaves.

*N*-acetylornithine (C00437; *m*/*z* 219.09755) is an acetylated biosynthetic intermediate of amino acids. Upregulated *O*-acetyl-L-homoserine (C01077; *m*/*z* 161.06476) is an acetylated L-homoserine involved in cysteine and methionine metabolism. Branched-chain organic acid 3-methyl-2-oxobutanoic acid (C00141; *m*/*z* 161.04424) was characteristically upregulated in leaves. Pantothenate (C00864; *m*/*z* 218.10237), biotin (C00120; *m*/*z* 259.07559), phylloquinone (C02059; *m*/*z* 529.27026), and delta-tocopherol (C14151, *m*/*z* 423.32561) were upregulated vitamin compounds in *S. cheesmaniae* leaves. Porphyrin metabolites, as exemplified by porphobilinogen (PBG) (C00931; *m*/*z* 271.09418), protoporphyrin (C02191; *m*/*z* 621.27057), pyropheophorbide *a* (C18064; *m*/*z* 579.26227), pheophorbide *a* (C18021, m/z 591.25991), and red chlorophyll catabolite (C18022, *m*/*z* 625.26542) were found upregulated in infected *S. cheesmaniae* leaves. Porphyrins as photosynthesizers are reported to exert substantial efficiency to kill plant pathogens. Likewise, compounds 3-beta-hydroxyergosta-7,24(24(1))-dien-4alpha-carboxylate (C22119, *m*/*z* 423.32561), 26-hydroxycastasterone (C19873, *m*/*z* 525.34338), typhasterol (C15793, *m*/*z* 529.27026), teasterone (C15791, *m*/*z* 529.27026), and brassinolide (C08814, *m*/*z* 525.34338) involved in brassinosteroid biosynthesis were identified as upregulated potential metabolites.

### 3.6. Metabolite Biomarkers/Pathways for Resistance Response in S. cheesmaniae

**Figure 10:**
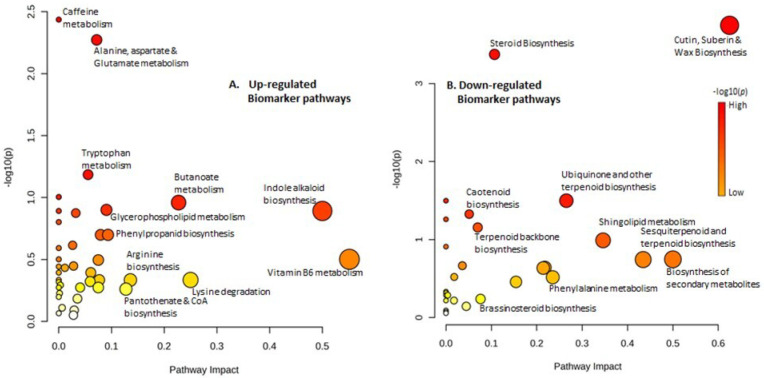
Pathway impact analysis of up- (**A**) and down-regulated (**B**) biomarker pathways in *S. cheesmaniae*.

According to FC ≥ 2, VIP score ≥ 1, and *p* < 0.05 values, 34 up- and 41 downregulated “significantly different metabolite features” were identified as “biomarkers”. Upregulated biomarker compounds included 3-hexenol, 5-phosphoribosylamine, 5-hydroxykynurenine, kaur-16-en-18-oic acid, 5-phosphoribosylamine, 2-isopropylmaleate, indolepyruvate, tetradecanoic acid, pantothenate, and O-acetyl-L-homoserine (Table 1). Downregulated biomarker metabolites were represented by pheophytin *a*, zymosterol, episterol, sphinganine, obtusifoliol, lariciresinol, etc. (Table 2). Upregulated metabolite biomarkers were critically mapped with vitamin B6 metabolism, indole alkaloid biosynthesis, and other metabolic pathways (Figure 10A). Interestingly, the downregulated metabolite biomarkers were mapped upon pathways such as biosynthesis of cutin, suberin and wax biosynthesis, secondary metabolites, phenylalanine, carotenoids, brassinosteroids, and terpenoids (Figure 10B), which are categorically known to be modulated to favor plant defense, suggesting their prominent role in plant survival.

## 4. Discussion

Tomato is a model horticultural crop plant for studying cellular, biochemical, and molecular responses linked to plant growth and development under biotic stress conditions [22]. However, mechanisms underlying plant responses in leaves of moderately disease-resistant wild tomato *S. cheesmaniae* after *A. solani* interaction in terms of metabolite profile and metabolic pathways have not yet been investigated. Untargeted metabolomics indicate differential metabolite profiles and biosynthetic pathways as plant responses against stress and aid to identify key functional biomarker metabolites [23]. Such a comprehensive approach enables metabolite profiling to analyze complex plant metabolic responses, products, and processes to identify crucial defense-related biosynthetic pathways and elucidate secondary metabolic networks [24]. Metabolite profiling of *S. cheesmaniae* plants reflected clear differences and revealed high metabolite variability in a number of metabolite features (9451 in MCTR vs. 5911 in MCNTR) and their abundance. Comparative functional analysis identified 3371 metabolite features with KEGG identifiers involved in various biosynthetic pathways. The FC (≥2.0) analysis revealed 541 significantly upregulated and 485 downregulated metabolite features annotated with the *S. lycopersicum* compound library in the PLANT CYC PMN database. A total of 34 upregulated and 41 downregulated metabolite biomarkers were identified by OPLS-DA analysis in the leaves of *S. cheesmaniae* under pathogen-challenged conditions.

The enrichment analysis classified upregulated metabolites to fatty-acid conjugates, aldehydes, pyridine carboxaldehydes, tryptamines, indolyl carboxylic acid, indoles, phenols, chalcones, straight-chain fatty acids, vitamins K and E, biotin, pyridoxals, phosphoethanolamines, and brassinosteroids. These metabolite groups play a prominent role in plant defense against diseases. Fatty-acid derived compounds (C-16 and C-18) actively act as signals and modulate effector-triggered systemic immunity [25]. Aldehydes, especially volatile compounds, activate defense genes and resistance responses against pathogenic fungi [26], while pyridine carboxaldehydes are typically involved in vitamin B6 biosynthesis [27], which induces resistance against pathogens while promoting plant growth [28]. Significantly upregulated tryptamine (C00398, *m*/*z* 219.11339) minimizes fungal infection-induced damage and regulates the biosynthesis of serotonin (5-hydroxytryptamine) [29], which is a signaling molecule for stress response mechanisms in plants. The indolyl carboxylic acid group that includes indole-3-carboxylic acid derivatives are secondary metabolites which accumulate inside the cell wall in response to pathogen interaction [30], reflecting their defensive role against biotic conditions [31]. Indoles [32], phenols [33], and flavonoids including chalcones [34] have immense direct applications in plant defense, with an indirect involvement in plant growth. Straight-chain fatty acids [25], pyridoxal, vitamins K and E, and biotin [35] are antioxidants with a potential role in minimizing stress-induced damage due to ROS and improving plant development under stress [36]. Phosphatidylethanolamines (LPE) are natural phospholipids that coordinate defense responses, interfere with oxidative bursts, and improve basal immunity against pathogens [37]. Brassinosteroids play a specific role in signaling in plant–microbe interaction [38]. Phenylethylamines (PEA), tyramine, and serotonin are plant-derived monoamines identified as upregulated metabolites. All significantly upregulated compound groups directly or indirectly improve plant fitness against stress conditions and are essentially involved in growth–defense tradeoffs [39]. We expect that their abundant accumulation might have aided plants to adapt to disease stress.

Among downregulated compound groups, we reported cyclic alcohols, prenylated hydroquinones, sphingosines and sphinganines, ergostane steroids, catecholamines, beta keto acids, and phenethylamines. Cyclic secondary alcohols are often oxidized to ketones having prominent biological properties [40]. Prenylated hydroquinones exert a strong antioxidant effect [41]. Structural membrane components such as sphingolipids (sphingosines and sphinganines) act as signal molecules in cell functions against infectious bacterial and fungal pathogens [42,43]. Downregulation of these compound groups in *S. cheesmaniae* may have significance toward lowering plant resistance.

Porphyrins and their intermediates are crucial for ROS-mediated stress responses and for regulating complex networks that control stress-responsive genes [44]. In pathogen-challenged *S. cheesmaniae* plants, porphobilinogen, protoporphyrin, pheophorbide *a*, red chlorophyll catabolite, and pyropheophorbide *a* were identified as prominently upregulated metabolites of porphyrin metabolism. Porphobilinogen is a distant precursor of vitamin biosynthesis, protoporphyrin is involved in the biosynthesis of chlorophyll a, and pheophorbide *a* synthesizes red chlorophyll catabolite and pyropheophorbide *a*. Although porphobilinogen, chlorophyll catabolite pheophorbide *a*, and red chlorophyll catabolite pyropheophorbide *a* are reported to confer defense in organisms against different abiotic stressed conditions [45,46], their multifold overaccumulation in *S. cheesmaniae* leaves in response to the pathogen is supposed to play a prominent defensive role. Accumulation of pheophorbide *a* was reported to induce programmed cell death under darkness and illuminated conditions in transgenic *Arabidopsis* plants [47].

Hormones influence plant performance under biotic stresses. Plant hormones such as salicylic acid (SA), abscisic acid (ABA), jasmonic acid (JA), indole-acetic acid (IAA), and gibberellic acid (GA), alongside several other natural molecules such as brassinosteroids and strigolactones, regulate biotic stress signaling and mediate transcription factors (TFs) to regulate multiple signaling transduction pathways for plant defense against pathogenic challenges [48]. Prominent upregulation of IAA, ABA, JA, GA, GA derivatives and intermediates, brassinolide, and zeatin and its derivative as phytohormones was observed in the pathogen-challenged leaves at various levels. Diversified abundance of these compounds is believed to play a coordinated role in the plant hormone signaling cascade leading to protection against pathogens alongside the regulation of plant growth and development in *S. cheesmaniae* [48,49].

Plant–microbe interactions notably lead to multifold changes in polyamine metabolism of the host, making the interaction dynamic and complex, with profound changes in free and conjugated polyamines inside the tissues [50]. Upregulated *N-*acetylornithine, a compound of acetyl amino acids, biosynthesizes polyamines that mediate plant interactions with the pathogens. Adjio et al. [51] reported enhanced accumulation of *N-*acetylornithine in wildtype *Arabidopsis* plants under pathogen *P. syringae* infection. Overaccumulation of *O*-acetyl-L-homoserine, as reported in MCTR, may play a role in enhancing plant immunity against pathogens [52]. We presumed that all of these annotated over-accumulated metabolites in MCTR may have acted in a coordinative way to offer moderate protection in *S. cheesmaniae* against *A. solani*.

Cell-wall strengthening upon pathogen invasion is the strategic mechanism of protection in plants. Upregulation of compounds such as coniferyl alcohol and 5-hydroxyconiferyl alcohol in *S. cheesmaniae* leaves in diseased conditions may signify their role in lignin synthesis, through which plants create a physical barrier against pathogen invasion [53]. Polyunsaturated fatty acids (PUFAs) are precursors of key metabolites which mediate crosstalk between organisms and further serve as substrates to oxylipins, i.e., JA, which plays crucial role in defense against diseases [53]. Icosapentaenoic acid, a long-chain fatty acid, is a powerful elicitor that showed upregulation. The short-chain C6-alcohol 3-hexenol is an indispensable carbonyl volatile compound that offers aroma to tomato due to its unique grassy green characteristics, as well as induces defense responses in plants against diseases [54].

Vitamins as natural inducers of disease resistance [55] and carotenoids as photoprotective and antioxidant agents [56,57] are widely reported. Canthaxanthin, a red-hot keto-carotenoid pigment exerts potential antioxidant and free-radical-scavenging properties [58]. Oxygen-containing lutein and violaxanthin are pigments with stress-suppressing properties [59]. Xanthoxin, an apo-carotenoid sesquiterpenoid, is an intermediate in the biosynthesis of the plant hormone abscisic acid [59]. Phoenicoxanthin, a natural xanthophyll of carotenoid biosynthesis, plays a role in protection against oxidative stress [60]. Likewise, tocopherol, pyridoxine, and biotin induce resistance in plants against diseases, likely mediated via their antioxidant effect against generated ROS [61]. All these compounds showed a multifold increase in MCTR, offering defensive functions in *S. cheesmaniae* plants.

Flavonoids and brassinosteroids also showed multifold overaccumulation in pathogen-challenged plant leaves. Flavonoids, having the widest distribution as secondary metabolites, primarily respond to biotic stresses in plants [62]. Upregulated flavonoids included pinocembrin (5,7-dihydroxyflavanone), pinocembrin chalcone, and apiforol, a tetrahydroxyflavan that influences plant defense by mediating pathogen signaling responses, membrane permeability, quorum sensing, and pathogen virulence [63]. Chalcones phloretin and isoliquiritigenin are strong antioxidants that showed an increase [64]. Multifold upregulated brassinosteroid compounds 3-beta-hydroxyergosta-7,24(24(1))-dien-4alpha-carboxylate, 26-hydroxycastasterone, typhasterol, teasterone, and brassinolide in pathogen-challenged leaves play a prominent role as structural components of cell membranes and aid against plant stress [65]. Overaccumulation of sulfur-containing natural compounds such as 1,2-dihydroxy-5-(methylthio)pent-1-en-3-one, an aliphatic sulfide, may indicate a versatile role in plant resistance against pathogen infection [66].

Our study revealed evidence of pathogen-induced metabolic changes in *S. cheesmaniae* moderately resistant to *A. solani* and provided specific clues of metabolic pathways that play a crucial role in conferring moderate resistance toward pathogens. Pathogenic infection often shifts the secondary metabolite profile in plant tissues due to induced defense programs to confer various levels of changes in primary and secondary metabolism conferring resistance against diseases [67]. In addition to elucidating the impact of the pathogen on secondary metabolites and their pathways, which have been widely studied [68], we also critically analyzed the impact of infection on crucial metabolites and pathways linked to porphyrins, hormones, vitamins, carotenoids, and amino-acid and fatty-acid metabolism. Key upregulated biomarker metabolites revealed from this study included 5-phosphoribosylamine, 5-hydroxykynurenine, kaur-16-en-18-oic acid, pantothenate, and *O*-acetyl-L-homoserine. The predictive identification of these biomarkers in *S. cheesmaniae* independent of genetic or other climatic variation can be used to distinguish diseased and non-diseased conditions inside plants, on the basis of which diagnostic tools could be developed. This approach may also aid in identifying mQTLs to be utilized in resistance-breeding programs under biotic stress conditions.

## 5. Conclusions

To support the hypothesis that the intrinsic metabolite composition and chemical diversity help wild tomato plants withstand stress challenges, untargeted metabolomics of pathogen challenged and unchallenged plants was performed. We reported metabolite changes associated with moderate resistance against *A. solani* in the leaves of wild species *S. cheesmaniae* grown under normal and pathogen-challenged conditions. Plant leaf metabolite profiles were significantly differentiated, and both normal and diseased plants were discriminated not only by the presence/absence of specific metabolites as concluding factors, but also on the basis of the relative abundance of metabolites as important distinguishing criteria. Annotation of the metabolite features using the KEGG *S. lycopersicum* compound database led to the annotation of 3371 metabolite features with KEGG identifiers, which were enriched in a number of metabolic/biosynthetic pathways, mainly including secondary metabolites, cofactors, steroids, terpernoids, fatty acids, and brassinosteroids. Significantly upregulated (541) and downregulated (485) compounds distributed in different metabolite classes play a crucial role in defense, infection prevention, signaling, plant growth and development, and survival maintenance under challenged conditions. It is hypothesized that these metabolites individually and/or cumulatively influence plant responses against pathogenic interactions and provide protection against infection on a wider scale. To our knowledge, this study is the first holistic comprehensive metabolite profiling of *S. cheesmaniae* plants underlying *A. solani* infection, which led to the identification of metabolite biomarkers and their metabolic pathways. The results hold promise for developing disease-diagnostic tools based on key biomarker metabolites. The biomarker-based disease-resistance metabolic traits/biosynthetic routes could contribute to the development of mQTLs for supporting future biotic stress-breeding programs in tomato.

## Figures and Tables

**Table 1:** Up-regulated putatively identified metabolite biomarkers * on the basis of FC ≥ 2, VIP ≥ 1, and *p* < 0.05.

S. No.	Mass *(m*/*z)*	Matched Compound	Putatively Identified Biomarker Metabolites *	Fold Change	VIP Score (OPLS DA)
1.	229.03	C03090	5-Phosphoribosylamine	11.99	1.22
2.	278.12	C00864	Pantothenate	8.98	1.02
3.	203.06	C02631	2-Isopropylmaleate	7.51	1.34
4.	203.06	C00331	Indolepyruvate	7.50	1.34
5.	161.04	C00164	Acetoacetate	6.27	1.59
6.	161.04	C00232	Succinate semialdehyde	5.63	1.59
7.	229.20	C06424	Tetradecanoic acid	4.41	1.22
8.	162.03	C15606	1,2-Dihydroxy-5-(methylthio)pent-1-en-3-one	4.32	1.58
9.	228.08	C00881	Deoxycytidine	4.21	1.23
10	220.10	C00590	Coniferyl alcohol	3.8	1.26
11.	243.09	C00588	Choline phosphate	3.39	1.17
12.	257.10	C00670	sn-Glycero-3-phosphocholine	3.29	1.12
13.	203.05	C00331	Indolepyruvate	3.27	1.35
14.	187.02	C00121	D-Ribose	3.21	1.43
15.	219.11	C00398	Tryptamine	2.99	1.27
16.	219.10	C00437	N-Acetylornithine	2.99	1.27
17.	247.02	C00018	Pyridoxal phosphate	2.92	1.15
18.	191.03	C00438	N-Carbamoyl-L-aspartate	2.79	1.40
19.	261.04	C05817	(1R,6R)-6-Hydroxy-2-succinylcyclohexa-2,4-diene-1-carboxylate	2.65	1.09
20.	145.09	C08492	3-Hexenol	2.62	1.19
21.	271.09	C00931	Porphobilinogen	2.59	1.06
22.	169.05	C13482	Phosphodimethylethanolamine	2.47	1.54
23.	259.04	C00352	D-Glucosamine 6-phosphate	2.36	1.11
24.	257.06	C09762	Liquiritigenin	2.32	1.12
25.	277.12	C00449	N6-(L-1,3-Dicarboxypropyl)-L-lysine	2.29	1.02
26.	119.03	C00033	Acetate	2.29	1.12
27.	163.06	C06001	(S)-3-Hydroxyisobutyrate	2.28	1.57
28.	146.04	C00423	trans-Cinnamate	2.24	1.16
29.	168.05	C00436	N-Carbamoylputrescine	2.24	1.55
30.	229.03	C03090	5-Phosphoribosylamine	2.18	1.23
31.	261.11	C00140	N-Acetyl-D-glucosamine	2.15	1.09
32.	247.13	C13455	Abscisic aldehyde	2.14	1.14
33.	259.08	C00120	Biotin	2.14	1.11
34.	231.05	C16361	1,3,7-Trimethyluric acid	2.13	1.22

* Compounds putatively annotated with Plant CYC PMN database of *Solanum lycopersicum* are reported.

**Table 2:** Down-regulated putatively identified metabolite biomarkers * on the basis of FC ≥ 2, VIP ≥ 1, and *p* < 0.05.

S. No.	Mass *(m*/*z)*	Matched Compound	Putatively Identified Biomarker Metabolites *	Fold Change	VIP Score (OPLS DA)
1.	429.37	C01753	beta-Sitosterol	0.403	1.89
2.	419.08	C00448	trans,trans-Farnesyldiphosphate	0.079	1.84
3.	413.34	C15777	Episterol	0.297	1.87
4.	399.33	C05437	Zymosterol	0.001	1.76
5.	609.27	C18098	Primary fluorescent chlorophyll catabolite	0.002	1.75
6.	565.41	C08579	Antheraxanthin	0.002	1.75
7.	318.21	C00909	Leukotriene A4	0.009	1.75
8.	337.24	C06427	(9Z,12Z,15Z)-Octadecatrienoic acid	0.019	1.75
9.	357.26	C19616	18-Hydroxyoleate	0.002	1.73
10	565.39	C08583	Canthaxanthin	0.003	1.67
11.	293.21	C18218	16-Hydroxypalmitate	0.005	1.67
12.	417.34	C15882	2-Methyl-6-phytylquinol	0.007	1.66
13.	489.39	C02477	alpha-Tocopherol	0.004	1.65
14.	417.34	C15882	2-Methyl-6-phytylquinol	0.008	1.63
15.	434.27	C05797	Pheophytin a	0.009	1.59
16.	439.36	C22121	Cycloeucalenone	0.011	1.58
17.	329.27	C01530	Octadecanoic acid	0.007	1.57
18.	311.22	C04717	(9Z,11E)-(13S)-13-Hydroperoxyoctadeca-9,11-dienoic acid	0.43	1.57
19.	339.16	C20693	Carlactone	0.321	1.56
20.	380.35	C08281	Docosanoic acid	0.172	1.54
21.	549.41	C06098	Zeaxanthin	0.011	1.54
22.	423.36	C01943	Obtusifoliol	0.011	1.47
23.	337.04	C00364	dTMP	0.441	1.46
24.	324.10	C01762	Xanthosine	0.374	1.44
25.	180.10	C05332	Phenethylamine	0.032	1.44
26.	491.21	C05427	Phytyldiphosphate	0.012	1.42
27.	411.38	C00751	Squalene	0.213	1.37
28.	344.28	C02934	3-Dehydrosphinganine	0.131	1.36
29.	282.28	C00836	Sphinganine	0.432	1.26
30.	198.08	C02631	2-Isopropylmaleate	0.441	1.23
31.	337.20	C01226	12-OPDA	0.431	1.22
32.	324.20	C19691	Farnesylcysteine	0.206	1.21
33.	309.19	C05819	Menaquinol	0.314	1.18
34.	383.10	C20693	Carlactone	0.031	1.17
35.	344.28	C02934	3-Dehydrosphinganine	0.043	1.12
36.	427.14	C01674	N,N-diacetylchitobiose	0.434	1.11
37.	217.05	C00794	D-Sorbitol	0.251	1.11
38.	453.33	C15800	3-Dehydro-6-deoxoteasterone	0.011	1.09
39.	493.28	C18217	16-Feruloyloxypalmitate	0.031	1.09
40.	423.36	C01943	Obtusifoliol	0.032	1.06
41.	441.07	C10646	Lariciresinol	0.491	1.02

* Compounds putatively annotated with Plant CYC PMN database of *Solanum lycopersicum* are reported.

## Data Availability

The data presented in this study are available on request from the corresponding author. The data are not publicly available due to privacy.

## Supplementary Materials

The following supporting information can be downloaded at: https://www.mdpi.com/article/10.3390/metabo13050585/s1, Table S1: Supplementary Materials.
